# Advances in dietary pattern analysis in nutritional epidemiology

**DOI:** 10.1007/s00394-021-02545-9

**Published:** 2021-04-25

**Authors:** Christina-Alexandra Schulz, Kolade Oluwagbemigun, Ute Nöthlings

**Affiliations:** Department of Nutrition and Food Sciences, Nutritional Epidemiology, University of Bonn, Endenicher Allee 19b, 53115 Bonn, Germany

**Keywords:** Dietary pattern, Hypothesis based, Exploratory, Gut microbiome, Metabolome

## Abstract

**Background and Purpose:**

It used to be a common practice in the field of nutritional epidemiology to analyze separate nutrients, foods, or food groups. However, in reality, nutrients and foods are consumed in combination. The introduction of dietary patterns (DP) and their analysis has revolutionized this field, making it possible to take into account the synergistic effects of foods and to account for the complex interaction among nutrients and foods. Three approaches of DP analysis exist: (1) the hypothesis-based approach (based on prior knowledge regarding the current understanding of dietary components and their health relation), (2) the exploratory approach (solely relying on dietary intake data), and (3) the hybrid approach (a combination of both approaches). During the recent past, complementary approaches for DP analysis have emerged both conceptually and methodologically.

**Method:**

We have summarized the recent developments that include incorporating the Treelet transformation method as a complementary exploratory approach in a narrative review.

**Results:**

Uses, peculiarities, strengths, limitations, and scope of recent developments in DP analysis are outlined. Next, the narrative review gives an overview of the literature that takes into account potential relevant dietary-related factors, specifically the metabolome and the gut microbiome in DP analysis. Then the review deals with the aspect of data processing that is needed prior to DP analysis, particularly when dietary data arise from assessment methods other than the long-established food frequency questionnaire. Lastly, potential opportunities for upcoming DP analysis are summarized in the outlook.

**Conclusion:**

Biological factors like the metabolome and the microbiome are crucial to understand diet-disease relationships. Therefore, the inclusion of these factors in DP analysis might provide deeper insights.

## Introduction

Dietary pattern (DP) analysis has long been established in nutritional epidemiological research [1, 2]. DPs are examined to characterize dietary behavior achieving a resemblance of real life, in which foods (and nutrients) are eaten in combination. They offer a close relation to dietary recommendations on the food level and account for the potential interplay of nutrients in whole diets, which generally is not reflected in the single food group or nutrient approaches. DP, covering the complexity of dietary intakes, are then most often investigated with respect to their relation to disease risk and contribute to the generation of evidence for disease prevention [3].

The approaches in dietary pattern analysis can be grouped into three categories [3–6]: the hypothesis-driven approaches, the exploratory approaches and hybrid approaches of the two. The hypothesis-driven approach relies on prior information based on current knowledge regarding defined dietary components and their relation to health promotion and/or to major diet-related-diseases. Hypothesis-driven DPs arise from allocating points to the predefined dietary components using a scoring system. The scores, also called dietary indices, reflect diet quality or adherence to national dietary guidelines (Healthy Eating Index, etc.), or are based on hypotheses on healthful dietary habits such as the Dietary Approaches to Stop Hypertension (DASH) diet [7], or the Mediterranean (MED) diet [8].

The exploratory approaches derive DPs without any hypotheses. Exploratory approaches traditionally derive DPs solely based on the underlying dietary data whereby a larger set of dietary variables is aggregated and reduced to form a smaller set of variables determining the DPs. The DPs describe the variation in dietary intake in a population based on correlations between nutrients, food items or food groups as reported in the respective dietary assessment instruments [3]. This approach usually employs simple to extensive statistical analysis to achieve its aim. The most widely adopted methods are cluster analysis and principal component analysis (PCA) [9] and the most often derived exploratory patterns in Western populations are commonly given simplistic interpretation of “Western DP” or “Prudent DP” [9]. Typically, a Western DP is characterized by greater intakes of white bread, red meat, processed meat, potatoes, and high-fat dairy products. In a Prudent DP, greater amounts of fruits, vegetables, whole grains, poultry and fish are consumed [9].

Since 2003, the hybrid methods that help to explain the relationship of diet to health via intermediate factors such as the reduced rank regression (RRR) have more widely been used [10, 11]. RRR takes into account prior knowledge about variables potentially relevant for the pathophysiological consequences of dietary intake, and thus takes a priori knowledge into account. With respect to the grouping of food items, however, it is exploratory by design, making it a hybrid approach.

Nutritional epidemiological research questions and methodology are evolving and high-dimensional biomarker data are increasingly integrated. Given these expansions, the scope of traditional DP approaches may be limited. Indeed, this includes both conceptual and statistical limitations. Since DPs could be a reflection of broader lifestyle patterns beyond diets [12], incorporating potentially relevant dietary-related factors such as sociodemographic, socioeconomic as well as genes and biological factors that are not assumed to be dependent on dietary intake may be necessary. Recently, a DP was generated to account for the contributions of sex, race/ethnicity, and body mass index [13]. To extend these thoughts, hypothesis-driven dietary patterns lately have also expanded by taking environmental aspects into account [14–16]. Moreover, non-traditional biological factors such as the metabolome and the gut microbiome, which are closely associated with dietary intake, have only limitedly been accounted for in DP analysis. Improvements have also been made in exploratory methods that derive DPs solely based on reported dietary intake, because the most commonly used exploratory method, the convectional PCA involves several but crucial subjective decisions, and the resulting DPs are sometimes challenging to interpret. Some of the novel exploratory methods that addresses these limitations include Treelet transform analysis [17] and the Gaussian graphical models [18]. Similarly, the RRR, for instance, can handle multiple response variables, thus implying the use of a number of metabolites (derived from metabolomics) or taxa as response variables. But, RRR being linear regression model based is not suitable when the functional relationship to model between dietary variables and the dependent variable is nonlinear [19]. Besides, RRR essentially treats dietary data as independent variables.

A crucial step in utilizing dietary data for DPs, regardless of the aforementioned approaches per se, is dietary data preprocessing. The extent of the data preprocessing depends on the assessment instrument used to generate the dietary data and on the statistical methods to be applied. The food frequency questionnaire (FFQs) is the conventional dietary assessment instrument in large epidemiological studies, and data from FFQs have been traditionally used to derive DP. However, prone to bias, nowadays the use of multiple short-term instruments potentially complemented by an FFQ have been recommended [20], which leaves more granular dietary data to reflect dietary intake. Besides, nutritional epidemiological research has evolved to incorporate novel tools to assess dietary intake [21]. These novel tools may have an impact on the form and utility of dietary intake in DP analysis. Therefore, these advances call for both conceptual and statistical adaptations and advances in DP analysis.

The current review aims to pick up some of the recent developments and strategic thoughts into dietary pattern analysis. Particularly, we attempt (1) to give an overview of complementary or novel hypothesis-driven and exploratory approaches to derive DPs, and highlight their uses, peculiarities, strengths, limitations, and scope; (2) to review the literature that takes into account potential relevant dietary-related factors, specifically the metabolome and the gut microbiome in dietary pattern analysis; and (3) to give an overview of the literature using dietary data from dietary assessment methods other than FFQ to compose DPs.

## Recent advances in hypothesis-driven dietary pattern analysis

The centerpiece of a priory index is the underlying information used to derive the DP. Healthful dietary habits are usually based on an interpretation of the overall literature relating diet to health [9]. The most commonly known indices take into account either cultural aspects or a certain health condition. Thus, existing hypothesis-driven indices are based on either food-based dietary guidelines, traditional eating habits of a specific geographical region, disease prevention, or the broader lifestyle. An overview of selected dietary indices and their rationalities, components and scoring systems is given in Table 1.

**Table 1:** Selected examples of dietary indices

Rationale	Score	Hypothesis based on	Dietary components, recommended intake, and scoring system
Food-based dietary guidelines	Healthy Eating Index (HEI-2015) [25]	2015–2002 Dietary Guidelines for Americans	5/10* points each if complying to the recommended servings below Total fruits: ≥ 0.8 c equivalents/1000 kcal Whole fruits: ≥ 0.4 c equivalents/1000 kcal Total vegetables: ≥ 1.1 c equivalents/1000 kcal Greens and beans: ≥ 0.2 c equivalents/1000 kcal Whole grains*: ≥ 1.5 oz equivalents/1000 kcal Dairy*: ≥ 1.3 c equivalents/1000 kcal Total protein foods: ≥ 2.5 oz equivalents/1000 kcal Seafood and Plant Protein: ≥ 0.8 oz equivalents Fatty acids*: (PUFAs + MUFAs)/SFAs ≥ 2.5 Refined grains*: ≤ 1.8 oz equivalents/1000 kcal Sodium*: ≤ 1.1 g/1000 kcal Added sugar*: ≤ 6.5% of energy Saturated Fats*: ≤ 8% of energy TOTAL HEI-2015 score = 0–100
Region origin	Mediterranean (MED) [8]	Adherence to the Mediterranean diet	5 points each if complying with the recommended servings below Non-refined Grains > 4/d Vegetables > 4/d Potatoes > 2/d Fruits > 3/d Full-fat dairy ≤ 10/wk Red meat ≤ 1/wk Fish > 6/wk Poultry ≤ 3/wk Legumes, nuts and beans > 6/wk Olive oil ≥ 1/d Alcohol < 300 mL/d but > 0 TOTAL MedDiet score = 0 to 55
Disease related	Dietary Approaches to Stop Hypertension (DASH) [7]	Recommended diet to prevent and treat high blood pressure [Joint National Committee on Prevention 2003] based on DASH-trails [41, 42]	1 point each if complying with the recommended servings below Total grains ≥ 7/d Vegetables ≥ 4/d Fruits ≥ 4/d Dairy ≥ 2/d Meat, poultry and fish ≤ 2/d Nuts, seeds and legumes ≥ 4/wk Total fat ≤ 27% of kcal Saturated Fat ≤ 6% of kcal Sweets ≤ 5/wk Sodium ≤ 2400 mg/d TOTAL DASH score = 0 to 10
	Mediterranean-DASH Intervention for Neurodegenerative Delay (MIND) [43]	Cross-method of the MED and DASH dietary pattern with an emphasis on linking the dietary components and servings to neuroprotection and dementia prevention	1 point each if complying with the recommended servings below Whole grains ≥ 3/d Green leafy vegetables ≥ 6/wk Other vegetables ≥ 1/d Berries ≥ 2/wk Red meat and products < 4/wk Fish ≥ 1/wk Poultry ≥ 2/wk Beans > 3/wk Nuts ≥ 5 /wk Fast/fried food < 1/wk Olive oil primary oil Butter, margarine < 1 T/d Cheese < 1/wk Pastries, sweets < 5/wk Alcohol/wine 1/d Total MIND score = 0–15
Lifestyle focused	Low-Risk Lifestyle Behaviors score [129]	Relationship between lifestyle behaviors on various health outcomes including cardiovascular disease, diabetes, all-cause mortality, and mortality from cancer [129]	1 point each if complying with the recommendation below Never smoked or less than 100 cigarettes smoked during life time Healthy diet = top 40% of the Healthy Eating Index of the population [129] Adequate physical activity = being moderately or vigorously active, 5 and/or 3 times per week Moderate alcohol consumption = men ≤ 2 drinks/day and women ≤ 1drinks/day, respectively, but for both more than 0 drinks per month Total low-risk lifestyle behaviors score = 0–4

* indicates the following components where 10 points apply (i.e. Whole grain*; Dairy*; Fatty acids*; Refined Grain*; Sodium*; Added sugar*; and Saturated Fats*)

*d* day, *g* grams, *mg* milligrams, *wk* week, *PUFAs* polyunsaturated fatty acids, *MUFAs* monounsaturated fatty acids, *SFAs* saturated fatty acids

### Food-based dietary guidelines focused

Various dietary indices have been introduced to assess diet quality [1, 2, 22] or adherence to dietary recommendations [1]. The Healthy Eating Index (HEI), developed by Kenneth et al. to assess adherence to the US Dietary Guidelines [23], was probably one of the first indices to measure how well national dietary recommendations were met. Since its creation in 1995, the HEI has been updated in correspondence to the most current version of the Dietary Guidelines for Americans at any time (i.e. HEI-2010 [24], HEI-2015 [25]). Moreover, an alternative HEI (aHEI) has been developed. As an alternative, this DP is based on foods and also nutrients that have been consistently associated with lower chronic disease risk [26]. Furthermore, the Dietary Guidelines for Americans Adherence Index (DGAI) has also been developed in reference to the Dietary Guidelines for Americans [27]. The concept of creating dietary indices based on national recommendations has been transferred to several dietary guidelines (e.g. Danish, French, Swedish and German recommendations) [28–31].

### Regional origin

Knowledge about differences in death rates across populations from different geographical regions relating to dietary intake, in particular mono- and saturated fat consumption, goes back to the “seven countries study” by Ancel Keys [32]. The traditional Mediterranean diet, a DP found in Italy and Greece in the 1950s and 1960s, was found to be associated with a lower risk of coronary heart disease [32]. Since there was a great interest in investigating DP from different geographical regions and indices have been developed which capture geographical eating patterns. Well-known examples are the MED [33, 34] or Nordic [35] dietary indices, which focus on the traditional diet consumed in the Mediterranean and the Nordic countries. In addition, an alternative MED index (aMED) exists, which reflects an adaptation of the traditional MED [33] to non-Mediterranean countries [36]. Furthermore, some modified versions of the MED have been developed to adapt the MED to the countries such as Lebanon [37] and Japan [38], which differ in food culture compared to Greece where the MED was developed [33].

### Disease relationship oriented

The World Cancer Research Fund (WCRF) and the American Institute of Cancer Research (AICR) have released eight recommendations (plus two special recommendations) on diet, physical activity, and weight management for cancer prevention [39]. A composite score of these recommendations has been found to potentially lower the risk for most types of cancers [40] as well as death from respiratory diseases, death from circulatory diseases and, death from cancer [16]. In addition, the DASH DP has been associated with health benefits particularly with lower blood pressure [41, 42]. Of note, also a cross-method of the Mediterranean diet and the DASH diet, called MIND (Mediterranean-DASH Intervention for Neurodegenerative Delay) exists, which emphasizes the dietary components and servings linked to neuroprotection and dementia prevention [43].

### Lifestyle oriented

In line with the WCRF/AICR score [16], which includes several lifestyle factors in addition to dietary factors, some more recently developed scores that, in addition to diet, incorporate several modifiable lifestyle factors such as smoking, obesity and physical activity exist [44, 45]. This development goes back to the insight, that dietary behavior can also be seen as an element of lifestyles and links to further behavioral factors which have been shown to be associated with disease risk and prevention. A recent meta-analysis of 22 prospective observational studies reported a 66% reduced cardiovascular risk with adherence to several lifestyle behaviors [46]. This indicates there is a growing interest and relevance in the analysis of patterns going beyond the inclusion of dietary factors only.

## Complementary exploratory approaches in dietary pattern analysis

Exploratory DP analysis has been extensively reviewed in several pieces of literatures such as Moeller et al. [3]. Thus, in the current work, we focus on methods complementary to traditional ones, which have been developed in the last 15 years. The fact that the widely used exploratory method, the PCA has some shortcomings that make interpretation difficult, primarily necessitated the development of these complementary methods. While variants of PCA, such as sparse PCA that addressed some of the limitations of conventional PCA have been developed, other methods have emerged. These complementary approaches which can be broadly classified into latent class and clustering techniques have long found extensive application in a broad range of research fields, albeit they are new to DP analysis. The commonly used complementary approaches are presented below.

Treelet transform analysis (TT) is a latent class method that can be viewed as an amalgamation of PCA and hierarchical clustering analysis [17]. It provides the opportunity to describe DPs in multivariate dietary data with substantive meaning and interpretation. TT produces sparse components in an elegant and simple fashion by combining ideas from PCA and hierarchical clustering analysis. It also provides a concise visual representation of loading sparsity patterns and the general dependency structure of the data. Due to its sparsity (interpretability), a trade-off with explained variance of DPs becomes inevitable. Moreover, unlike PCA that relies on rules of thumb and ad hoc thinking for determining the number of DPs to retain, TT has a fast and efficient algorithm for determining the optimal number of DPs to be retained as well as assessing the stability of DPs. TT has been used to derive DP at the level of nutrients [47], food items [48] and food groups [49, 50]. The five DPs obtained from the intake into 39 food groups in a group of older German Adults were “alcohol and red meat”, “bread, margarine, and processed meat”, “fruiting vegetables and vegetable oils”, “tea and miscellaneous”, and “pasta and rice, and sauce”[50].

Another complementary method, the sparse latent factor models (SLFM) that are based on a Bayesian modelling framework aims to provide parsimonious relations between high-dimensional variables and latent factors by forcing less influential associations to have a zero association in the model [51, 52]. The only available literature on the application of sparse latent factor models in DP analysis was a recent paper on the intake of 102 food items in a group of young American adults [13]. The obtained seven DP were “fruit, nuts, several vegetables, fish and poultry”, “red and processed meat, potatoes, fried foods, pasta dishes, and breads”, “a cluster of snacks and sweets”, “a group of Hispanic foods”, “meat and dietary alternatives”, “alcoholic beverages”, and “cereal with milk” [13].

Gaussian graphical models (GGM) is an important exploratory analysis that identifies the conditional independence structure in the data set by assessing pairwise correlation between two variables controlling for other variables [18, 53]. GGM also introduces sparsity in generating DPs. A study that used GGM for DP analysis on 49 food groups identified clusters of red meat and cooked vegetables, dairy products, sweet foods, and fruits and vegetables [54].

The random forest with classification tree analysis (RF-CTA) is a clustering-based method. Random forest uses an algorithm to create multiple classification or regression trees based on a dependent variable of interest [55]. Subsequently, the classification tree analysis identifies mutually exclusive subgroups of a population that share common characteristics that influence the dependent variable of interest [56]. This method has been used to identify DPs comprising soy products, wine, fast foods, and French fries [57].

Independent component analysis (ICA) is another latent class method that finds a linear representation of non-Gaussian data so that the components are statistically independent or as independent as possible [58]. To our knowledge, ICA has not yet been applied in DP analysis; however, a report from the Nordic Research Council suggests that ICA would be very relevant because the DP that would be obtained will be independent of one another [19].

Preprocessing of input dietary variables such as dealing with missing variables and standardization (scaling or centering) are issues that should be addressed before using some of these approaches. Methods to handle missing dietary variables include multiple imputations [59] and mixed models [60]. Standardization is encouraged because it ensures that mean and the variance of each dietary variable have no influence on the obtained DPs [3]. It should also be noted that since exploratory DPs are data-driven, DPs might not be reproducible across populations or even within the same population over time [3]. Additionally, to ensure widespread applicability, it is necessary that these approaches are available across the well-known statistical software packages. More importantly, statistical principles of these approaches should be well understood since the default settings may not always give optimal solutions. Obviously, there are advantages and disadvantages of these complementary methods with their respective practical worth and repercussions. Table 2 provides an overview of the features/aims, peculiarities, strengths, and limitations of TT, SLFM, GGM, and RF-CTA in DPs analysis.

**Table 2:** Selected complementary exploratory dietary pattern analysis approaches

Exploratory dietary pattern approach	Features/aims	Peculiarity	Strengths	Limitations	References
Treelet transform analysis (TT)	TT is a cross-method of principal component analysis and hierarchical clustering analysis	1. Forces sparse DPs 2. Produces cluster tree that reflects local dependency relationships of the dietary variables as well as a coordinate system for the dietary data at each level of the cluster tree 3. Cluster tree level (cut-level) that must be chosen influences both the sparsity and the composition of DPs 4. Implemented as a Mata function in STATA statistical software	1. Uses the correlation or covariance matrix of dietary intake to derive DPs 2. TT procedure enables the selection of the number of DPs and a cut-level by a data-driven (cross-validation) method 3. Several functions to aid in model selection and output analysis 4. Stability assessment analysis by subsampling approach 5. Sparsity is optimal because a dietary variable is loaded on only one DP 6. DPs are potentially simpler to interpret 7. The latent variable is numerical so DP scores (continuous values) can be assigned to study participants	DPs are not independent	[17, 49, 50]
Sparse latent factor models (SLFM)	SLFM provide parsimonious relations between high-dimensional variables and latent factors by forcing less influential associations to have a zero association in the model	1. Uses the posterior inclusion probability obtained from the sparse latent factor analysis to determine food and DP pairs that have non-zero associations 2. A model-based solution for dealing with missing dietary data 3. Sparsity of DPs: sparsity-inducing priors is an integral part of SLFM 4. Implemented in software for Bayesian factor regression models	1. Interpretability of DPs is achieved by its sparsity and inclusion threshold of dietary variables showing a significant and a posterior inclusion probability > 0.95 for a given DP 2. Several approaches to infuse biological or non-dietary variables	Sparsity is not optimal because a dietary intake variable may be loaded on one or a few DPs	[13, 51]
Gaussian graphical models (GGM)	GGM identifies conditional independence structure in dietary variables by assessing pairwise correlation between two variables, while controlling for effects of the other variables in the network model	1. Selects a model on rank-based dietary variable 2. Estimates a single DP as a unique solution for the estimated model 3. Sparsity of DPs depends on a regularization parameter that can be derived using different criteria 4. Implemented in R statistical software	1. It can also be used for ordinal data or data comprising a mixture of categorical and continuous dietary variables 2. Stability assessment analysis	Requires Gaussian-distributed data The latent variable is categorical so DP scores (continuous values) cannot be assigned to study participants Spurious relationships may affect network interpretation A single DP may not fully exploit the multivariate dietary data sets	[18, 53, 54]
Random forest with classification tree analysis (RF-CTA)	RF-CTA belongs to the family of classification trees. This approach generates many decision trees that are constructed using different sets of randomly selected dietary variables	1. Intuitive approach: easy to implement and understand 2. Produces decision trees that can be visualized 3. Implemented in R and other statistical software	1. It is a highly accurate classifier which produces an internal unbiased estimation of the generalization error 2. The model is robust to outliers 3. No standardization or scaling required prior to the analysis 4. Ability to handle missing values	The latent variable is categorical so DP scores (continuous values) cannot be assigned to study participants Less transparent Highly data dependent	[55–57]

## The metabolome and the gut microbiome in dietary pattern analysis

The metabolome and gut microbiome are expected to unlock the ‘black box’ in chronic disease epidemiology [61–63]. Although their causal relationship with most health conditions has yet to be confirmed, the metabolome and the gut microbiome are likely to improve our understanding of the link between DPs and health conditions. Therefore, there is a need to integrate the metabolome and the gut microbiome in DP analysis. A number of epidemiological studies associating DPs with the metabolome and the gut microbiome, particularly bacterial taxonomic composition appeared in the literature over the last ten years [50, 64–86]. These primary studies were retrieved from the PubMed (MEDLINE) database (terms: dietary patterns/indices, gut microbiome, and metabolome, and limited to articles published in English). We reviewed the references of retrieved studies for additional studies. These studies include those associating hypothesis-driven and exploratory DPs with single metabolites and metabolite patterns or scores as well as hypothesis-driven and exploratory DPs with single metabolites and metabolite patterns or scores. A summary of these studies is provided in Table 3.

**Table 3:** Studies associating dietary patterns with the metabolome and the gut microbiome

	Single metabolites	Metabolite patterns or scores	Single bacterial taxa	Population groups of bacterial taxa
Hypothesis-driven dietary patterns (DPs)	Four DPs related to the ratio of 363 metabolites [64], Two DPs related to 4369 metabolites [68], Four DPs related to 116 metabolites [71], Mediterranean DP associated with three short-chain fatty acids [72], four diet quality indexes associated with 1316 metabolites [74], four DPs related to several metabolites [76], Patterns of meat and seafood consumption related to 42 metabolites [77], Mediterranean DP associated with 1165 metabolites [78], Mediterranean DP associated with 59 metabolites [81], Mediterranean DP associated with 175 metabolites [84]	Mediterranean DP associated with a metabolite score [84]	Three DPs related to 26 bacteria [70], Mediterranean diet associated with several bacteria [72], Mediterranean DP and Healthy Food DP related to several taxa [73], Two DPs related to several bacterial taxa [87]	Three DPs associated with enterotypes [81], unprocessed foods and processed food groups associated with phylogenetically grouped bacterial taxa [86], Two DPs related to two enterotypes [87]
Exploratory DPs	Seven PCA-derived DPs related to ratio of 363 metabolites [64], Three Cluster analysis-derived DPs related to plasma fatty acid profiles and metabolomic data [65], Seven PCA-derived DPs related to 163 metabolites [67], sparse PCA-derived meat and vegetable DPs associated with 130 metabolites [81]	Five TT-derived DPs related to eight TT-derived metabolite patterns [50], DPs explaining a maximum variation in the concentration of the seven classes of chemically similar metabolites were derived by RRR [66], Two PCA-derived DPs correlated with two PCA-derived metabolite patterns [74], RRR-derive DP associated with branched chain amino acids [79], RRR-derive DP associated with 853 metabolites [82]	Three cluster analysis-derived DPs related to seven bacteria [69], factor analysis-derived DPs associated with several bacteria taxa [85], Three PCA-derived DP associated with 24 bacterial taxa [86] two DPs were related to several bacteria taxa [89]	Five TT-derived DPs related to seven TT-derived groups of bacteria [50]

Evidence exists regarding the relation of Mediterranean DP with metabolites and microbiome. As opposed to the metabolome, findings from studies relating DPs, particularly exploratory DPs to the microbiome composition are few. While this may indicate that the interest in the role of the microbiome in DP is just emerging, it also suggests DPs shape the metabolome more than the microbiome. Nevertheless, the fact that recent studies have shown that the gut microbiome plays a role in the occurrence of several health conditions [63, 87], gut microbiome composition improves the prediction of glucose response [88], and the fact that a prudent DP was associated with a lower risk of *Fusobacterium nucleatum*-positive colorectal cancer [89] strengthens the relevance of the gut microbiome in DP analysis.

Irrespective of the DP approach or the number of metabolome or microbiome variables that were investigated, the approaches in the aforementioned studies can be broadly classified into two. The first is the two-stage approach where DPs are generated first and then they are related to single (or groups of) metabolites or single (or groups of) taxa in regression models. The second is a one-stage approach where metabolite-related or taxa-related DPs are generated using multivariable regression-based methods such as RRR. These two approaches are conceptually similar. The first approach has been applied to both the metabolome and the microbiome. On the other hand, the second approach using RRR has only been applied to the metabolome to generate metabolite-related DPs for seven classes of chemically similar metabolites [66], isoleucine, leucine, and valine [79], and six islet autoimmunity-associated metabolites [82].

The main goal of RRR is to generate DPs that capture as much variation in predicted response variables which are usually disease-related nutrients or biomarkers as such the focus is on the response variables. Therefore, RRR-generated DPs may not be diet behaviorally meaningful. This method would therefore be suitable when the goal of DP analysis is against the backdrop of exploring the effect of the combination of foods on health conditions that is mediated through the appropriately identified components of the metabolome and/or the gut microbiome. These components may be defined a priori [66, 79] or identified a posteriori [82]. As it has been explored for the metabolome, studies should use RRR or related methods to create DPs explaining disease or health outcomes microbiome signatures such as specific taxa or functions, broader microbiome compositions or functions, and microbiome diversity. The gut microbiome with its extensive and distinct metabolic repertoire complements the metabolic activity of the host enzymes by contributing microbial metabolites to the metabolome, either diet‐derived or from other sources [90]. Therefore, a pragmatic approach would be to generate DPs of disease-related microbial metabolites.

If a DP is related to a particular metabolite or taxon, there is a possibility that it is also related to other closely or distantly related metabolite(s) or taxa. Hence, an approach that maps the association of DPs with aggregated correlated metabolites or taxa would potentially yield higher statistical power. The study by Oluwagbemigun and colleagues underscores the importance of this approach where structured patterns of correlations present in each of the dietary, metabolome, and microbiome compositional domains were identified using TT and possible DP-metabolite patterns and DP-microbiome patterns associations were fitted in linear regression models [50]. This strategy is appealing because it fully explores the complex multivariate structure of the metabolome and microbiome data sets and ensures that grouping of metabolites is beyond chemical similarities or grouping of bacteria is beyond taxonomic relationships. This approach may be sufficient to reveal the potential relationships between few sets of dietary, metabolome, and microbiome variables that can be explored further. For instance, the single diets, metabolites or taxa contributing to each pattern can be used to generate new metabolome- or microbiome-related DPs using RRR or more parsimonious methods such as the partial least squares regression. Unlike the RRR, partial least squares regression (PLS) would generate DPs that have maximum covariance of both the dietary intake predictor variables and the metabolome and microbiome response variables.

In future, DP analysis that intends to incorporate the metabolome and/or microbiome should employ novel methods that can accommodate all high-dimensional predictor and response variables and simultaneously explore their associations and dependency patterns. Example of these novel methods, which have been applied in our research fields, includes the sparse PLS [91] and the sparse RRR [92]. The foregoing suggests that the additional information offered by the metabolome and the gut microbiome would be more exploited in exploratory DP when compared to hypothesis-driven DP analysis. Indeed, using a large part of the metabolome and the gut microbiome data and metabotyping [93] and enterotyping [94] populations into generally valid and biologically meaningful homogeneous subgroups would facilitate the integration of the metabolome and the gut microbiome in DP analysis.

Despite the optimism and the advantages of incorporating the metabolome and microbiome in DP analysis, their huge biological complexities and the methodological variation associated with generating metabolome and microbiome data suggest that translating metabolome- or microbiome-related DPs into practice may be challenging.

## Implication of dietary assessment methods for dietary pattern analysis

In the past, dietary data used for DP analysis was most commonly assessed by long-term dietary assessment instrument, FFQs [49, 95–99]. FFQs include informative food items consumed regularly, contributing substantially to the nutrient(s) of interest and ability to rank individuals according to their intake [9]. Different procedures for selecting informative food items have been described [9]. Fewer studies have used short-term instruments such as dietary recalls and dietary records [100–103]. All assessment methods do have their strengths and limitations. Thus, more recently it has been discussed that combining short-term and long-term dietary assessment instruments is a superior approach to derive usual dietary intake estimates in large-scale studies [20]. Whereas the combination of these instruments is promising, some challenges for DP which will be addressed below cannot be excluded.

Prior to DP analysis, food items are usually grouped into food groups. The main purpose of such grouping is to reduce the complexity of the obtained data. The number of food groups composed reported in the literature range from 25 [104] to 99 [102]. Most commonly the groups are formed according to their macronutrient composition (e.g., fat or fiber content), nutritional similarities, or culinary use [105–108]. Further rationalities for grouping food items include related food and drinks [102, 109], nutrient profile and consumption in the cohort under investigation [110], and their hypothesized contribution to diet–disease relationships [100, 111]. Additionally, some foods are oftentimes grouped into a separate group such as eggs, butter, margarine, pizza, soup, coffee, tea, or garlic [105, 108, 112]. Allocating a minimum of 5–10 participants per food group was another approach mentioned in the literature [113] based on the recommendation by Kass and Tinsley [114]. Decisions of aggregating food items may also be based on expert knowledge and experience. The extent to which different aggregation of food items from the same population and assessment instrument affect DP solutions are questions that should be considered in future studies.

At first, it needs to be considered that the number of food items reported in different populations may vary immensely. This may be on the one hand due to the variety of the diet consumed by a population under investigation, but also due to the applied dietary assessment instruments. Applying the instruments simultaneously over a similar period may be beneficial. FFQs are designed to capture the habitual dietary intake and include a list of defined food and drinks. Over a specific period in the past (commonly the previous year), the participants are asked to indicate their typical frequency of consumption. Depending on the purpose of the FFQ, these questionnaires include up to a few hundred selected food items, commonly ranging from, e.g. 20 items in “screeners” to 200 items for fully comprehensive lists intended to assess the total diet [115] (Table 4). Whereas, data from dietary recalls and records capture either dietary intake from the recent past (usually previous 24 h or 48 h) or are completed by participants at the time of consumption throughout a specific period of time (often 3–7 days). Portion sizes may be either weighted or estimated (non-weighted), brands (if relevant), and preparation (e.g. cooking method, the addition of fat, recipe ingredients, etc.) may also be reported [115]. Both of the latter tools are open ended in contrast to the pre-defined list of food items captured via FFQs. Thus, by the usage of a dietary recall and dietary record, the number of items reported in a study population may easily add up to a couple of thousand items (e.g. from the literature > 3500 [102], 4850 [116], and 7444 [117]).

**Table 4:** Characteristics of dietary assessment tool

	Characteristics	Number of food items
FFQ	Retrospective, usually concerning the previous year, both quantitative and semi-quantitative versions exist	List of predefined food items and food groups; usually < 300
Diet recall	Retrospective, usually previous 24 h or 48 h	Individually based the food intake recorded by the participant; open-ended; > 1000 items
Dietary record	Filled in during actual food intake, both weighted and unweighted	Individually based the food intake recorded by the participant; open-ended; > 1000 items

Thus, the more the detailed information arises from dietary recalls and records the more the effort might be required by the investigators to cluster the food items into food groups prior to analysis, given that much more items appear in the database which need to be sorted into groups. Still, FFQ-derived DP has been shown to be in general comparable with those derived from dietary record data in both children [118] and adults [108, 119]. Having access to much more detailed dietary data such as multiple FFQs, more recalls or recording days and variety of foods and the extent of their processing however may potentially offer the opportunity that more detailed food groups can be formed and thus more in-depth hypotheses can be tested. It has been discussed for instance that the preparation method of meat may play a role in the disease relationship [120–122] or that fermented dairy products may have a different disease association than non-fermented dairy products [123, 124].

## Summary and implications for future research

This review summarizes the most recent advances in DP analysis, which has been an established analytic approach in nutritional epidemiology. DP analysis has advanced the field of nutritional epidemiology by combining nutrients and foods and thus taking into account their synergistic effects [1, 2]. The three approaches, (a) hypothesis based, (b) data-driven, and (c) a hybrid approach of both, have been widely used during the last two decades. A relationship between diets and the metabolome and gut microbiome is strongly hypothesized. Recent developments in biomedical sciences, particularly advances in omics technologies, have made the quantification of several metabolites and the composition of a large number of gut bacteria feasible. This has made the inclusion of metabolome and gut microbiome in DP analysis relevant, necessary, and important, and calls for adequate statistical approaches. Lastly, the advancements in assessments of dietary data were also picked up in this review. The availability of more detailed data will allow defining food groups customized to the scope of specific research questions.

The strength of this review is that it provides a state of knowledge of DP analysis with a focus on novel approaches. To our knowledge, at writing, this review was the first to cover the potential inclusion of the metabolome and the gut microbiome in DP analysis. The major weakness of this review article is that some relevant studies might have not been evaluated. However, this review is comprehensive, each aspect was objectively introduced, and our inferences mostly adhere to the cited evidence.

The perspective of nutritional epidemiology has nowadays become more comprehensive and studies have a more holistic approach beyond the sole focus on the associations of dietary intake and disease risk. This widening of perspective addresses the linking of additional aspects related to diets in exposure definitions on the one hand, and the disentangling of underlying mechanisms between dietary intake and disease risk on the other hand. The latter is of importance for the general mechanistic understanding, but might also support the development of dietary disease prevention recommendations stratified for population groups. DP analysis can be instrumental in both directions. Advanced statistical approaches in DP analysis enable the incorporation of metabolome and gut microbiome to foster a thorough understanding of disease risk mechanisms and serve potential stratifications with respect to susceptible population groups. Finally, DP analysis should be adapted to the advancements in dietary assessment methods with respect to detail and granularity. Lately, DP very recently started to take effects beyond health outcomes into account and implications in relation to sustainability and environmental impact have come into focus. Yet, only very few studies have addressed sustainability aspects in exploring DP to-date [14, 15, 125–128]. Thus, there is a strong need to investigate the broader impact of diets on the environment and link these to disease prevention, for which DP analysis has the means to respectively expand its scope and close the gap of analyses of sustainable DPs with chronic disease risk.

Considering the above-mentioned advances in DP analysis would offer progressive insights into diet–disease relationships.

## Conclusion

DP analysis has revolutionized the field of nutritional epidemiology long ago making it possible to take into account that foods are consumed in combination and thus may have synergistic effects. In the recent past, new aspects relevant for DP analysis have emerged both conceptually as well as methodologically. Thus, future DP analysis should take additional aspects such as diet sustainability and environmental impact into account, to advance the capture of diet as a whole. Additionally, incorporating the metabolome and microbiome in DP analysis might help to reveal necessary insights into the diet–disease relationships helping targeted disease prevention through dietary efforts in the long run.
